# Bifunctional regulators of photoperiodic flowering in short day plant rice

**DOI:** 10.3389/fpls.2022.1044790

**Published:** 2022-10-20

**Authors:** Changhui Sun, Changcai He, Chao Zhong, Shihang Liu, Hongying Liu, Xu Luo, Jun Li, Yuxiu Zhang, Yuting Guo, Bin Yang, Pingrong Wang, Xiaojian Deng

**Affiliations:** ^1^ State Key Laboratory of Crop Gene Exploration and Utilization in Southwest China, Sichuan Agricultural University, Chengdu, China; ^2^ Rice Research Institute, Sichuan Agricultural University, Chengdu, China

**Keywords:** flowering, photoperiod, bifunctional regulators, critical day length, phytochrome, circadian clock

## Abstract

Photoperiod is acknowledged as a crucial environmental factor for plant flowering. According to different responses to photoperiod, plants were divided into short-day plants (SDPs), long-day plants (LDPs), and day-neutral plants (DNPs). The day length measurement system of SDPs is different from LDPs. Many SDPs, such as rice, have a critical threshold for day length (CDL) and can even detect changes of 15 minutes for flowering decisions. Over the last 20 years, molecular mechanisms of flowering time in SDP rice and LDP Arabidopsis have gradually clarified, which offers a chance to elucidate the differences in day length measurement between the two types of plants. In Arabidopsis, CO is a pivotal hub in integrating numerous internal and external signals for inducing photoperiodic flowering. By contrast, *Hd1* in rice, the homolog of *CO*, promotes and prevents flowering under SD and LD, respectively. Subsequently, numerous dual function regulators, such as phytochromes, *Ghd7*, *DHT8*, *OsPRR37*, *OsGI*, *OsLHY*, and *OsELF3*, were gradually identified. This review assesses the relationship among these regulators and a proposed regulatory framework for the reversible mechanism, which will deepen our understanding of the CDL regulation mechanism and the negative response to photoperiod between SDPs and LDPs.

## Introduction

The earth’s rotation around its tilt axis is the primary principle of external forces in the world, which repeatedly manifests in diurnal cycles and seasonal fluctuations. Plants have relied on exact and regular variations in day length to trigger the right flowering time (Imaizumi and Kay, 2006; Amasino, 2010; Andres and Coupland, 2012; Anwer and Davis, 2013; Sun et al., 2021). Garner and Allard, in the 1920s, discovered that many plants flowered at the proper time independent of their sowing dates, which was first described as photoperiodic phenomenon (Garner and Allard, 1920; Garner and Allard, 1923). Then, photoperiod is acknowledged as a crucial pathway for flowering time (Song et al., 2015). According to different responses to photoperiod, plants were divided into short-day plants (SDPs), long-day plants (LDPs), and day-neutral plants (DNPs) (Thomas and Vince-Prue, 1996; Izawa, 2007). While LDPs prefer longer days for flowering and DNPs flower regardless of day length, SDPs flower earlier on shorter days. According to physiological investigations, SDPs have a different day length measurement system from LDPs (Thomas, 1998). However, what causes the difference in their photoperiodic response remains unclear.

**Figure 1:**
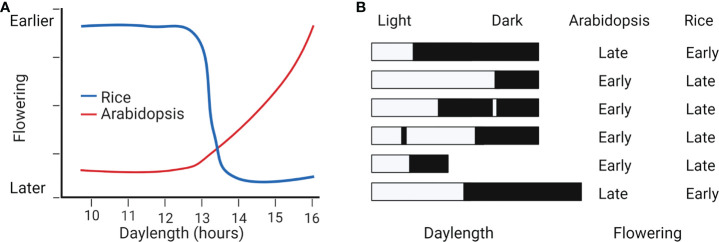
Different photoperiod and NB responses between SDP rice and LDP Arabidopsis. **(A)** Opposite responses to photoperiod in rice and Arabidopsis. Flowering in Arabidopsis gradually becomes earlier with increasing day length. However, rice has a ≈13.5 h CDL and detects changes of 15 minutes for flowering transition. **(B)** Effect of dark length to flowering in rice and Arabidopsis. Rice flowering is early in the long dark. Arabidopsis flowering is early in long day or short dark. NB treatment induces flowering in Arabidopsis but suppresses flowering in rice (Thomas and Vince-Prue, 1996).

In photoperiodic flowering, the critical threshold for day length (CDL) refers to the photoperiod that distinguishes between flowering and vegetative growth. LDP Arabidopsis does not detect any CDL and flowers steadily earlier as the day length gets longer (Izawa, 2007; Wang et al., 2021b). However, SDP rice has a 13.5 h CDL and can even detect changes of 15 minutes daily to decide when to flower (Thomas and Vince-Prue, 1996; Nishida et al., 2002; Itoh et al., 2010; Sun et al., 2021) (**Figure 1A**). Furthermore, SDPs observed the dark period’s length rather than the day’s length. This conclusion was reached after observing a night-break response phenomenon (NB). Many SDPs, but not LDPs, are significantly inhibited from flowering during the night by a light pulse (Hamner and Bonner, 1938) (**Figure 1B**). Therefore, the CDL and NB are two important indicators for detecting differences in day length response between SDPs and LDPs.

Two main photoperiodic measurement models have been developed due to decades of physiological study into how plants detect day length. According to the external coincidence model, a photo-inducible phase of the circadian cycle and the illuminated portion of the light signal must coincide for photoperiodic responses to be elicited. In this model, light has two distinct effects, training the circadian system and causing photoperiodic responses during the photo-inducible phase of the cycle. According to the internal coincidence model, the internal consistency of circadian rhythms is the only way to induce the photoperiodic response (Bunning, 1936).

In the past 20 years, the flowering molecular mechanisms in rice and Arabidopsis have gradually clarified, which offers a chance to ascertain the molecular difference in day length measurement between the two types of plants (Izawa, 2007). Numerous studies suggest that functional differences in *CONSTANS* (*CO*) and *Heading date 1* (*Hd1*) play essential roles. *Hd1* is the first bi-functional flowering gene that was found in rice. Subsequently, numerous bi-functional regulators, such as phytochromes, *Grain Number, Plant Height and Heading Date 7* (*Ghd7*), *Days to Heading 8* (*DTH8*), *Oryza sativa Pseudo-Response Regulator 37 OsPRR37*), *Oryza sativa GIGANTEA* (*OsGI), Oryza>sativa LATE ELONGATED HYPOCOTYLN* (*OsLHY)*, and *Oryza sativa EARLY FLOWERING 3* (*OsELF3*), were identified. Bi-functional regulator here is defined as the effectors that could promote and inhibit flowering in different day length conditions (such as *Hd1*), as well as the modifiers (such as *Ghd7*, *DTH8*) that could modify the effectors and affect their dual functions (**Table 1**). However, no bi-functional flowering genes were found in Arabidopsis. It was proposed that the reversal mechanism is crucial to investigating the CDL regulation mechanism and will fill in our understanding of the negative response to photoperiod between SDPs and LDPs (Yano et al., 2001; Izawa, 2007). This review assesses the relationships between these dual function regulators and proposes a putative regulatory framework for the reversible mechanism in rice.

**Table 1:** Regulatory factors of reversal mechanism in rice heading date.

Gene Name	MSU ID	Materials	SD heading	LD heading	Function	Reference
Hd1	LOC_Os06g16370	hd1	Later	Earlier	Effector	Yano et al., 2000
Ghd7	LOC_Os07g15770	ghd7	Earlier	Earlier	Modifier	Xue et al., 2008
DTH8	LOC_Os08g07740	dth8	Later	Earlier	Modifier	Du et al., 2017
OsPRR37	LOC_Os07g49460	osprr37	Earlier/Later	Earlier/Later	Modifier/Effector	Koo et al., 2013; Zhang et al., 2019; Hu et al., 2021
SE5	LOC_Os06g40080	se5	Earlier	Earlier	Modifier	Izawa et al., 2002
PHYA	LOC_Os03g51030	phya	WT	Minor Later	Modifier/Effector	Takano et al., 2005
PHYB	LOC_Os03g19590	phyb	Earlier	Earlier	Modifier	Takano et al., 2005
PHYC	LOC_Os03g54084	phyc	WT	Earlier	Modifier	Takano et al., 2005
OsGI	LOC_Os01g08700	OsGI-RNAi	Later	Earlier	Effector	Hayama et al., 2003
OsLHY	LOC_Os08g06110	oslhy	Earlier	Later	Effector	Sun et al., 2021
OsELF3	LOC_Os06g05060	OsELF3-OX	Later	Earlier	Effector	Yang et al., 2013
Hd6	LOC_Os03g55389	hd6	WT	Earlier	Modifier	Nemoto et al., 2018
Hd16	LOC_Os03g57940	hd16	Later	Earlier	Modifier/Effector	Nemoto et al., 2018
OsCCT22	LOC_Os06g19444	oscct22	Later	Earlier	Effector	Zhang et al., 2021
OsCCT38	LOC_Os11g05930	oscct38	Later	Earlier	Effector	Zhang et al., 2021
OsCCT41	LOC_Os12g16160	oscct41	Later	Earlier	Effector	Zhang et al., 2021
OsVIL1	LOC_Os08g12430	osvil1	Later	Earlier	Effector	Zhao et al., 2011; Jeong et al., 2016

## Photoperiodic flowering pathways in arabidopsis and rice

### *CO* is a hub in photoperiodic flowering in Arabidopsis

**Figure 2:**
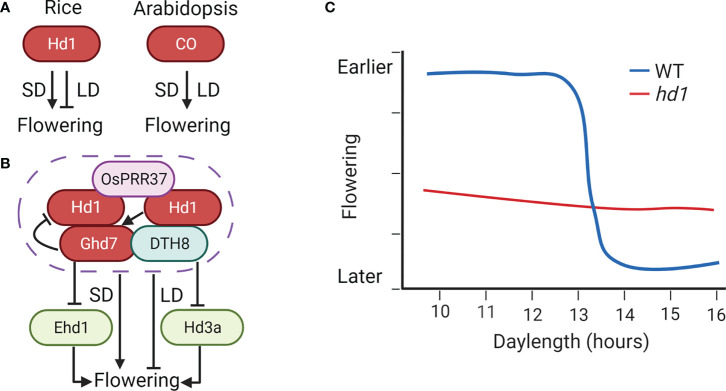
Bifunctional *Hd1* and its enhancers *Ghd7*, *DTH8*, and *OsPRR37* in rice. **(A)** The opposite function between *Hd1* and *CO* under LD. **(B)** Three enhancers of bifunctional *Hd1*. *Ghd7*, *DTH8*, and *OsPRR37* could improve the dual function of *Hd1*, thus enhancing the photosensitivity of flowering in rice. **(C)** *Hd1* predominantly regulates photoperiodic response in rice. Arrows represent positive regulation, inhibitors represent negative regulation, and graphical overlap represents protein physical interaction.

The external coincidence hypothesis could partly explain the photoperiodic flowering in Arabidopsis (Imaizumi and Kay, 2006). The key flowering regulator *CO* is hypothesized to be a hub in integrating numerous internal and external signals into photoperiodic flowering (Shim et al., 2017) (**Figure 2A**). *CO* produces a B-box-type zinc-finger transcriptional activator that stimulates the florigen gene *FLOWERING LOCUS T* (*FT*) in a light-dependent manner (Yanovsky and Kay, 2002). *GIGANTEA* (*GI*) serves as a bridge between *CO* and the circadian clock by positively regulating the expression of *CO*. This *GI*-*CO*-*FT* pathway works as a key mechanism for daylength dependent flowering promotion pathway in Arabidopsis (Shim et al., 2017).

Furthermore, the internal circadian clock system sets the *CO* expression gate, and the light directly influences the stability and activity of CO protein (Yanovsky and Kay, 2003; Hayama and Coupland, 2004). Therefore, flowering time is controlled by the coincidence of light with a specific circadian phase defined by strong *CO* expression. Then, increased quantities of *FT* mRNA are created by this exogenous coincidence, which eventually encourages the transition from vegetative to reproductive growth (Yanovsky and Kay, 2002; Bouche et al., 2016).

### Unique photoperiodic flowering pathways in rice

In rice, a sophisticated network with more than 80 flowering genes has been investigated, and the molecular mechanism of day length recognition gradually surfaced (Cao et al., 2021; Zhou et al., 2021). Rice has two florigen genes, *Heading date 3a* (*Hd3a*) and *RICE FLOWERING LOCUS T 1* (*RFT1*) (Komiya et al., 2008). There are primarily two pathways for photoperiodic flowering, a unique LD flowering suppression route, *Ghd7*-*Early Heading Date 1* (*Ehd1*)-*Hd3a*/*RFT1*, and an *OsGI*-*Hd1*-*Hd3a*/*RFT1* flowering pathway, which is conserved to the *GI*-*CO*-*FT* pathway of Arabidopsis (Sun et al., 2014; Chen et al., 2022). Among them, both *Ehd1* and *Ghd7* are unique flowering regulators in rice. *Ehd1* encodes a B-type response regulator and forms homo-dimers to operate as a transcription factor (Doi et al., 2004; Cho et al., 2016). *Ehd1* promotes heading by upregulating the expression of *Hd3a* and *RFT1* in both long day (LD) and short day (SD), forming an *Ehd1*-*Hd3a*/*RFT1* module in rice (Chen et al., 2022). *Ghd7* encodes a CCT-domain transcription factor, prefers to express under LD and confers flowering repression (Xue et al., 2008; Itoh et al., 2010).

### CDL gates florigen gene *Hd3a* expression in rice

Different from *FT* in Arabidopsis, rice florigen gene *Hd3a* expression is gated by CDL, which is consistent with physiological research (Thomas and Vince-Prue, 1996; Nishida et al., 2002; Itoh et al., 2010; Sun et al., 2021). *Hd3a* expression is at fairly high levels in less than 13 hours (h) of day length conditions, but it drops off at day lengths of 13.5 h and becomes undetectable at day lengths of more than 14 h. Two unique mechanisms regulated by *Ehd1* and *Ghd7* are thought to control the CDL of *Hd3a*. When blue light corresponds with the morning phase defined by *OsGI*-dependent circadian clocks, *Hd3a* production is stimulated by *Ehd1* expression. On the other hand, *Ghd7* is acutely induced when phytochrome signals and photosensitive phases coincide, and this induction suppresses *Ehd1* expression the following morning (Itoh et al., 2010).

### Bifunctional *Hd1* and its enhancers in rice

*Hd1*, a homolog of Arabidopsis *CO*, is a significant quantitative trait locus (QTL) that predominantly regulates photoperiodic response in rice (Yano et al., 2000). Interestingly, *Hd1* serves in stimulating heading under SD and in repressing heading under LD, in contrast to the active role of *CO* (Yano et al., 2000) (**Figure 2A**). In certain genetic backgrounds of rice, *hd1* mutant almost loses the photosensitivity of flowering (**Figure 2C**). By contrast, this bifunctional phenomenon was not found in flowering regulators in Arabidopsis characterized so far (Bouche et al., 2016). Furthermore, the amount of *Hd1* mRNA was not significantly influenced by photoperiod changes, proving that the function of *Hd1* is certainly not at the transcription level. Therefore, post-transcriptional regulation may thus be the key to controlling the Hd1 function (Yano et al., 2000).

Many investigations revealed that *Ghd7*, *DTH8*, and *OsPRR37* could improve the dual function of *Hd1*, thus enhancing the photosensitivity of rice. *Ghd7*, *DTH8*, and *OsPRR37* are initially identified as flowering inhibitors and pleiotropic genes controlling plant height, heading date, and yield (Xue et al., 2008; Wei et al., 2010; Yan et al., 2011; Dai et al., 2012; Fujino et al., 2013; Koo et al., 2013; Liu et al., 2013; Yan et al., 2013; Gao et al., 2014; Du et al., 2017). Generally, *Ghd7* is crucial in converting *Hd1*, and *Ghd7* alone can determine the bi-function of *Hd1*. The protein complex generated by Ghd7-Hd1 proteins specifically binds to the cis-regulatory region of *Ehd1* (Nemoto et al., 2016). This protein-protein interaction between Ghd7 and Hd1 is also likely to suppress or impede the active ability of Hd1 (Zhang et al., 2017). DTH8 is a putative HAP3 subunit and directly interacts with Hd1 for the transcriptional repression of *Hd3a* (Wei et al., 2010; Du et al., 2017). But *DTH8* alone does not appear to determine the dual function of *Hd1*. Furthermore, by binding to the promoter region of *Ghd7*, DTH8 could form a protein complex with Hd1 to stimulate the transcription of *Ghd7* (Wang et al., 2019). Additionally, there is also a physical interaction between Ghd7 and DTH8, suggesting that they could control rice flowering synergistically (Li et al., 2015b) (**Figure 2B**). For *OsPRR37*, we will discuss it separately in the circadian clock section. Moreover, different haplotype combinations of *Hd1*, *Ghd7*, *DTH8*, and *OsPRR37* are important for photoperiodic adaptation in rice. In the Minghui63 background, *Ghd7* or *OsPRR37* reverse the *Hd1* function collectively rather than separately (Zhang et al., 2017). The greatest digenic interaction in the functional *Hd1* backgrounds was *Ghd7* by *DTH8* under LD, whereas *Ghd7* by *OsPRR37* under SD (Zhang et al., 2019; Zhou et al., 2021; Chen et al., 2022).

## Phytochromes

### The nature of phytochromes

**Figure 3:**
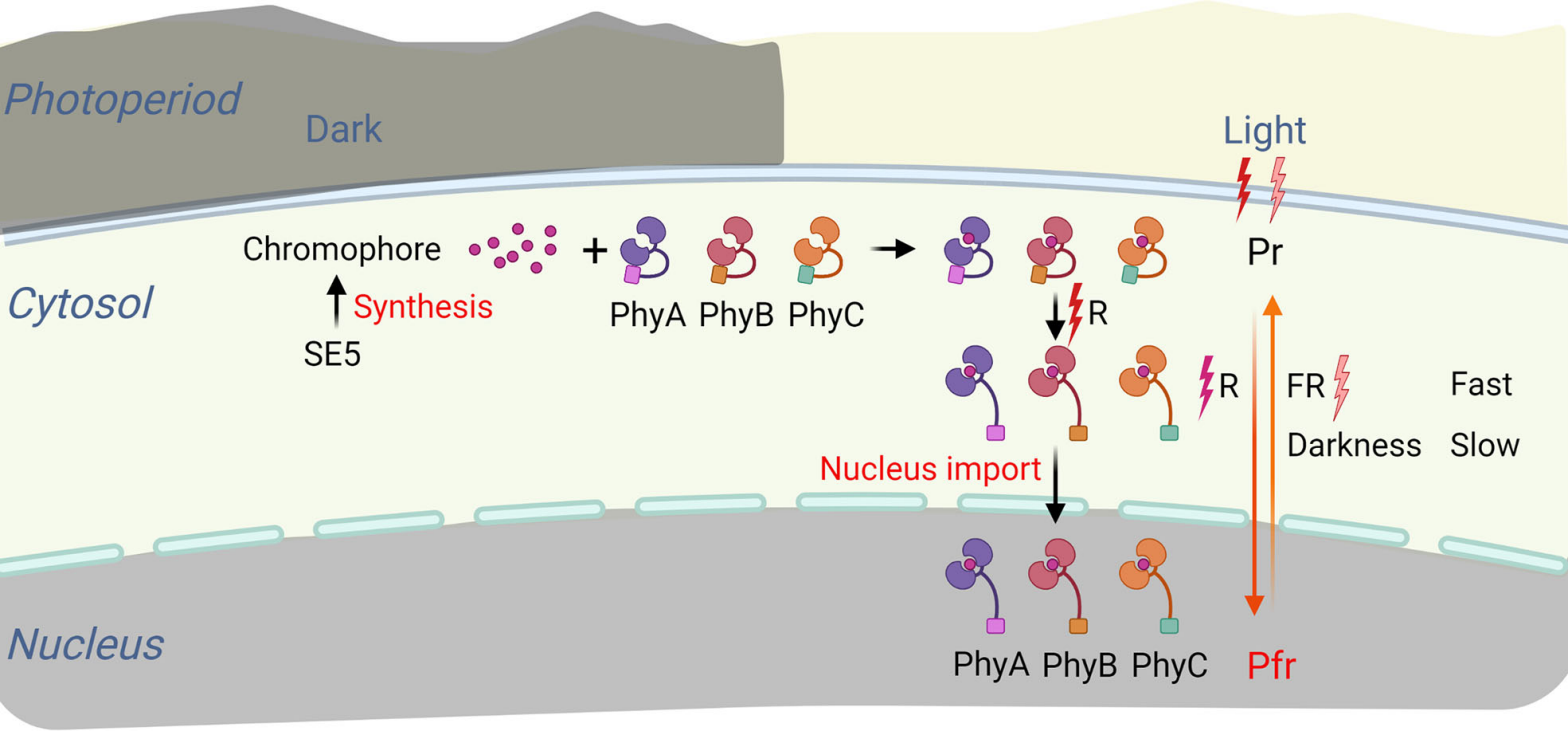
Phytochromes import to the nucleus after converting from inactive Pr form to active Pfr form. Phytochromes could change from inactive Pr to active Pfr under Red light (R). The active Pfr can be reverted to the inactive Pr by either a slow reaction caused by darkness or a quick response caused by exposure to far-red light (FR). When the inactive Pr form transforms into the active Pfr form, phytochromes leave their initial location (cytoplasm) and enter the nucleus.

The transmission of the initial photoperiodic signals to the final flower-forming element through three steps, light perceived, circadian clock, and signal output (Song et al., 2015). In the first step, light is perceived by various photoreceptors, such as phytochromes (red/far-red light receptor) and cryptochromes (blue light receptor) (Bae and Choi, 2008; Shim et al., 2017). Phytochromes are created as chromoproteins, where the apoprotein is joined to a billin chromophore. Exposure to red light (R) could change phytochrome conformations from inactive Pr to active Pfr. The active Pfr can then be transformed back to inactive Pr by either a gradual reaction caused by darkness or a rapid response caused by exposure to far-red light (FR) (Mancinelli, 1994; Quail, 1997; Fankhauser, 2001). Therefore, phytochromes can act as the developmental switch (Li et al., 2011). The inactive Pr form is initially localized in the cytoplasm and enters the nucleus after converting to the active Pfr form (Van Buskirk et al., 2012; Klose et al., 2015). In the nucleus, phytochromes interact with several regulatory components (such as PILs or PIFs) to control the expression of downstream genes and mediate light responses (Xu et al., 2015; Pham et al., 2018) (**Figure 3**).

In higher plants, phytochrome genes are encoded by a small family. The phytochrome in Arabidopsis consists of five members, PhyA to PhyE (Sharrock and Quail, 1989). However, only three phytochromes, PhyA, PhyB, and PhyC, exist in rice (Mathews and Sharrock, 1997) (**Figure 3**). The distinct phytochrome signal route between Arabidopsis and rice may be due to the presence or absence of PhyD/E (Pham et al., 2018). Phytochromes are also classified based on their stability in light and darkness. The PhyB to PhyE are all type II (light steady), whereas PhyA is a type I (light labile) (Li et al., 2015a). When exposed to R or white (W) light, PhyA levels rapidly decrease, but they are most plentiful in seedlings developed in the dark. PhyB is the most abundant phytochrome in plants cultivated in the light, while PhyC to PhyE are less abundant (Strasser et al., 2010; Hu et al., 2013; Cheng et al., 2021).

### Phytochromes and flowering

Takano (2005) did excellent work examining the function of all possible combinations of *PhyA* to *PhyC* single and double mutants in rice flowering. Depending on the day length, these mutants display various heading phenotypes (Takano et al., 2005). Under nature long day (nLD), *phyB* and *phyC* are engaged in decreasing flowering time, while *phyA* does not change the heading date. Double mutant *phyB phyC* flowers as early as *phyB* or *phyC* single mutant, demonstrating that the two phytochromes have a similar influence. Although *phyA* single mutant cannot alter the flowering, the double mutants *phyA phyB* and *phyAphyC* flower much earlier than *phyB* or *phyC*. These findings suggest that *phyA* mutation alone does not significantly affect flowering time. However, in the background of *phyB* or *phyC* mutants, *phyA* mutation significantly influences the flowering time under nLD (Takano et al., 2005).

Under SD, the *phyC* mutant displayed almost the same heading as the wild type, and the *phyA* mutant showed a minor later flowering, while the *phyB* mutant flowered earlier. *phyA phyC* and *phyB phyC* double mutants flowered simultaneously with those of the *phyA* or *phyB* single mutants, indicating that *PhyC* has no appreciable influence on the floral induction under SD. Interestingly, in contrast to earlier flowering in LD, *phyA phyB* double mutant flowered later in SD and even later than in LD, making it a simulated LDP (Takano et al., 2005). These findings suggest a close association between phytochromes and *Hd1* in the inversion mechanism of flowering regulation.

## *Hd1* and phytochromes

**Figure 4:**
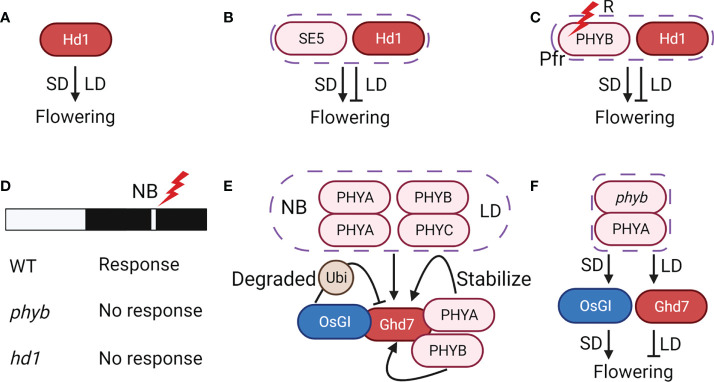
** **A strong connection exists between the reversal mechanism and phytochromes. **(A)** Hd1 alone is a flowering activator. **(B)** Hd1 changes to be a flowering suppressor with SE5 in LD. **(C)** Hd1 changes to be a flowering suppressor with PhyB in LD. **(D)** PhyB and Hd1 are critical to NB response. **(E)** Phytochromes, NB and LD regulate Ghd7 positively. OsGI degrades Ghd7 through the ubiquitination mechanism. **(F)** PhyA promotes the expression of OsGI under SD and Ghd7 under LD in phyb background. PhyA induces flowering under SD but represses it under LD without PhyB. Arrows represent positive regulation, inhibitors represent negative regulation, and graphical overlap represents protein physical interaction.

All phytochromes have the same chromophore. The chromophore is synthesized by the phytochromobilin-synthetic pathway, which is seriously damaged in an Arabidopsis mutant *hy1*. Therefore, the *hy1* mutant significantly reduces the function of the phytochromes (Cheng et al., 2021). *PHOTOPERIODI*C *SENSITIVITY 5* (*SE5*), a homolog of *HY1*, was the first flowering gene cloned in rice (Izawa et al., 2000). The mutant *se5* has less than 1% chromophore concentration, significantly hindering phytochrome production. As a result, *se5* has a very limited ability to send light signals for day length measurement and exhibits a very early flowering phenotype with no photoperiodic response, deservedly accompanied by the loss of CDL (Izawa et al., 2000) (**Figure 4A, B**).

Interestingly, *Hd1* constantly promoted flowering in *se5* under SD and LD, indicating that the functional conversion of *Hd1* depends on phytochromes (Thomas and Vince-Prue, 1996; Izawa et al., 2002; Andres et al., 2009) (**Figure 4A, C**). Further study showed that the dual function of *Hd1* that phytochromes switch may be involved in determining the CDL as the threshold for rice flowering (Izawa et al., 2000). Therefore, *Hd1* and phytochromes are both critical in sensing CDL, suggesting a strong link between them in regulating CDL.

Furthermore, experiments with non-24-hour light/dark cycles showed that *Hd1* serves as a circadian clock output. However, the circadian phase setting of *Hd1* expression is not affected in *se5* under SD and LD (Izawa et al., 2002). Therefore, the function convertible *Hd1* may be posttranscriptionally regulated by coincidence with different forms of phytochromes (Izawa et al., 2002). In the dark, Pfr could slowly transform into Pr (Elich and Chory, 1997; Eichenberg et al., 2000). According to End-Of-Day FR (EOD-FR) treatment observations, the Pfr form of *PhyB* could persist through the night and contribute to flowering inhibition under SD (Takano et al., 2005). Therefore, *Hd1* serves as a flowering repressor by interacting with the Pfr form. In contrast, *Hd1* activates flowering without this interaction. The overlap between *Hd1* and Pfr depends greatly on the photoperiod since the Pfr diurnal pattern differs from the *Hd1* expression rhythm. *Hd1* should have a larger overlap period under LD than under SD, thus making it a flowering gene with two distinct functions (Izawa et al., 2002) (**Figure 4C**). However, it has not been reported whether phytochromes could directly interact with Hd1. However, phytochromes could regulate the modifiers of Hd1 by protein interaction, such as Ghd7 and OsGI, thus affecting the function of Hd1 (Osugi et al., 2011; Zheng et al., 2019).

It seems that NB and CDL share a set of molecular mechanisms. Short exposure to light during the night (NB) imitates SD to LD and delays flowering in SDPs. However, different spectra of light have different NB effects. Red and blue light NB downregulated *Hd3a* expression, but far-red light NB did not. The impact of red light NB on *Hd3a* could be reversed by subsequent far-red light treatment. Interestingly, *phyB* mutants do not respond to NB, and their flowering time is not affected by NB, suggesting that *PhyB* is essential to NB (Ishikawa et al., 2009) (**Figure 4D**). Furthermore, *PhyB*-mediated flowering suppression by NB is *Hd1* dependent, which is also a primary cause of flowering suppression under LD (**Figure 4D**). However, rather than the change of *Hd1* expression controlled by the circadian clock, flowering is predominantly controlled by the direct action of light. As the Hd1 protein level is unaffected by light, protein activity but not the stability of Hd1 is the actual cause of photoperiodic flowering (Ishikawa et al., 2011).

## *Ghd7* and phytochromes

*Ghd7* mRNA levels increase as the day length becomes longer in the WT, but there are no appreciable changes in the *se5* mutant. Additionally, the expression of *Ghd7* was also strictly controlled by NB. Consequently, phytochromes might act as a mediator for light signals to induce *Ghd7* expression (Itoh et al., 2010). Since *Ghd7* expression could be induced in *phyB phyC* but not in *phyA phyC* or *phyA phyB* double mutants, *PhyA* alone is adequate to promote *Ghd7* expression (Osugi et al., 2011) (**Figure 4E**). However, *Ghd7* expression is inhibited in the *phyA phyC* or *phyA phyB* double mutants, proving that *PhyB* and *PhyC* could work collectively to generate *Ghd7* expression under *phyA* mutant background. Therefore, *Ghd7* expression can be induced by light signals sent by the *PhyA*/*PhyA* homodimer or the *PhyB*/*PhyC* heterodimer (**Figure 4E**). Despite the phytochrome action of entraining the circadian clock in rice, phytochromes do not set the *Ghd7* expression gate (Osugi et al., 2011).

## Circadian clock is critical to the inversion mechanism

### OsGI

**Figure 5:**
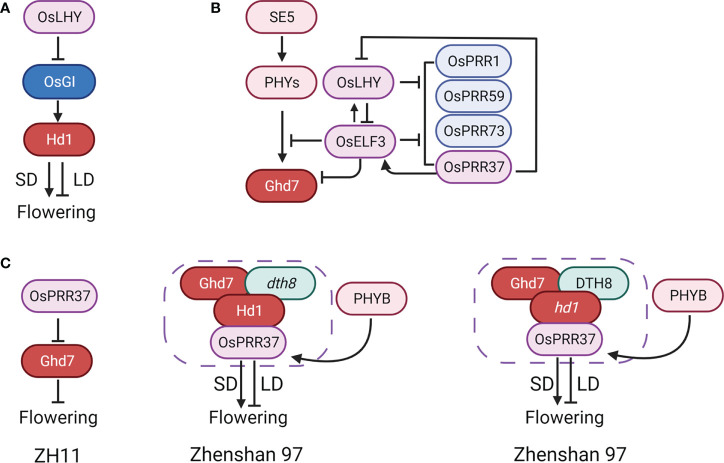
Circadian clock pathway is critical to the reversal mechanism. **(A)** The *OsLHY*-*OsGI*-*Hd1* pathway could fine-tune the CDL by adjusting the degree of coincidence between *Hd1* expression and day length. **(B)** The possible flowering mechanism of *OsELF3*. *OsELF3* suppresses the phytochrome signal, prevents light from entering *Ghd7* and impresses *Ghd7* expression. **(C)** *OsPRR37* acts as an activator or a suppressor depending on the background, and three genes, *Hd1*, *Ghd7*, and *DTH8*. Arrows represent positive regulation, inhibitors represent negative regulation, and graphical overlap represents protein physical interaction.

*GI* is identified as a flowering promoter in Arabidopsis. While, in rice, several studies of *OsGI* are not completely consistent. Early flowering under LD and late flowering under SD, similar to the *hd1* mutant, were caused by decreased *OsGI* expression, according to the first study using *OsGI* RNAi in the Norin 8 background (Hayama et al., 2003). A null mutant of *OsGI* by CRISPR/Cas9 method (cv. Nipponbare) flowered about 25 days earlier and confirmed this conclusion only in LD (Wang et al., 2021a). Therefore, *OsGI* might act upstream and control flowering in the same direction as *Hd1* (**Figure 5A**). However, *hd1 osgi*-*1* double mutants flowered intermediate between *hd1* and *osgi*-*1*, indicating that *OsGI* functions somewhat differently from *Hd1* (Izawa et al., 2011). In contrast, the *osgi*-*1* mutant (cv. Norin 8) prolonged flowering under SD, but did not show appreciable changes under LDs (Izawa et al., 2011). A T-DNA insertion mutant (cv. Dongjin) displayed an entirely distinct phenotype with null *OsGI* expression. It flowered 36 days later under SD (12 hours of light) and 9 days later under LD (14.5 hours of light) (Lee and An, 2015).

Furthermore, *OsGI* and phytochromes play antagonists in regulating *Ghd7* protein stability and flowering time. PhyA, PhyB and OsGI could directly interact with Ghd7, and PhyA and PhyB could inhibit the interaction between OsGI and Ghd7, thus helping to stabilize the Ghd7 protein (Zheng et al., 2019). *OsGI* is also a main distinct element between *PhyB* and *PhyA* downstream pathways, primarily activated by *PhyA* but unaffected in the *phyB* mutant (Lee et al., 2010). As mentioned above, *PhyA* induces flowering under SD but represses it under LD without other phytochromes (Osugi et al., 2011). One possible reason is that *PhyA* primarily affects the expression of *OsGI* under SD and *Ghd7* under LD in *PhyB* deficiency background (Lee et al., 2016) (**Figure 4F**).

### OsLHY

Recently, we demonstrated that *OsLHY* is a critical circadian rhythm gene in rice. The *oslhy* mutant delays flowering under LD but induces flowering under SD partly through the *OsGI*-*Hd1* pathway by binding to the CBS element in *OsGI* promoter (**Figure 5A**). Moreover, the CDL for *OsLHY* in *oslhy* (11-12 h) was prolonged in *oslhy osgi* double mutant (about 13.5 h), indicating the CDL set by *OsLHY* was *OsGI* dependent. Additionally, as the *oslhy hd1* double mutant headed simultaneously with *hd1* under both SD and LD, the reversible function of *OsLHY* entirely relied on *Hd1*. Therefore, the *OsLHY*-*OsGI*-*Hd1* pathway could fine-tune the CDL through the biological clock (Sun et al., 2021; Sun et al., 2022).

### OsELF3

The rice genome carries two *ELF3* homologs, *OsELF3* (*OsELF3*-*1*) and *OsELF3*-*2*. *OsELF3* can negatively regulate the expression of *OsPRR1*, *OsPRR37*, *OsPRR73*, and *OsPRR95*, while positively regulating the expression of *OsLHY* (Zhao et al., 2012; Itoh et al., 2019) (**Figure 5B**). Though *oself3*-*1* mutant flowered later under both SD and LD, the *OsELF3* overexpression transgenic plant showed 6 days earlier and 14 days later heading under LD and SD, respectively (Yang et al., 2013). It is consistent with earlier research about *Hd3*, which contains the *OsELF3* locus. *Hd3*-NIL plants with Kasalath allele locus shorten flowering time under SD and delay heading under LD (Lin et al., 2000). Then, *Hd3* was identified as two tightly linked loci, *Hd3a* and *Hd3b* (Monna et al., 2002). Further genetic evidence supports the assumption that the genes for *Hd17* (*OsELF3*-*1*) and *Hd3b* are located at the same locus (Matsubara et al., 2012).

The mechanism of flowering time regulation in *OsELF3* overexpression plants is still unclear. However, subsequent studies give some hints. Defective *OsELF3* did not appear to alter *Hd1* expression; however, its effect on flowering time vanished in *hd1* background, indicating that *OsELF3* may modulate the Hd1 protein activate through a post-transcriptional mechanism (Matsubara et al., 2012; Saito et al., 2012; Zhao et al., 2012). *OsELF3* also suppresses the phytochrome signal, prevents light from entering *Ghd7* and represses *Ghd7* expression (Saito et al., 2012). Recent research further proves this speculation. Compared with *se5*, the double mutant *se5 oself3*-*1* recovered *Ghd7* transcription and the photoperiodic flowering response. However, as *Ghd7* expression is not restored by *oself3*-*1* in the *phyAphyBphyC* triple mutant background, the triple mutant appears to be different from *se5* (Itoh et al., 2019) (**Figure 5B**).

### OsPRR37

*OsPRR37* encodes a pseudo-response regulator (PRR) protein, functions as a transcriptional repressor of clock genes, and delays flowering time through *Ehd1* in an expression level-dependent manner (Koo et al., 2013; Liu et al., 2013; Yan et al., 2013; Gao et al., 2014; Liu et al., 2018). However, *OsPRR37* could also function as a flowering promoter in cv. Zhonghua 11 (ZH11) background, mainly by inhibited *Ghd7* expression (Hu et al., 2021) (**Figure 5C**). Furthermore, *OsPRR37* could act as an activator or a suppressor depending on the status of three genes, *Ghd7*, *DTH8*, and *Hd1* (Zhang et al., 2019). For example, in the background of *Ghd7DTH8 hd1* and *Ghd7 dth8 Hd1* in Zhenshan97, *OsPRR37* promoted the heading under nature short day (nSD) but delayed the heading date under nLD (Hu et al., 2021). It has been reported that the *OsPRR37* (*DTH7*) transcript level was decreased in the *phyB* mutant, suggesting that *OsPRR37* might act downstream of *PhyB* (Gao et al., 2014). Further genetic analysis indicated that the function of *OsPRR37* required an intact *Ghd7*-related regulatory pathway, its upstream regulators *OsGI* and *PhyB*, and its interacting partner *Hd1* (Hu et al., 2021) (**Figure 5C**).

## Other bifunctional regulators

### *Hd6* and *Hd16*

**Figure 6:**
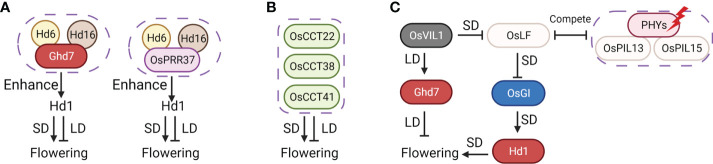
Other bifunctional regulators in rice. **(A)** *Hd6* and *Hd16* function as enhancers of the bifunctional action of *Hd1*, and fine-tune the CDL of flowering. **(B)** Three new dual-function flowering regulators in CCT domain-containing protein family. **(C)** *OsVIL1* could activate flowering by suppressing *OsLF* under SD and delay flowering by inducing *Ghd7* under LD. Arrows represent positive regulation, inhibitors represent negative regulation, and graphical overlap represents protein physical interaction.

Casein kinases (CKs) function in various eukaryotic signal transduction systems (Knippschild et al., 2005; Mulekar and Huq, 2014). In plants, CKI and CK2 are involved in phosphorylating circadian clock components and regulating flowering time (Sugano et al., 1998; Sugano et al., 1999). *Heading date 6* (*Hd6*) and *Heading date 16* (*Hd16*) encode CK2 alpha and CKI, respectively, are two flowering regulators in rice (Yamamoto et al., 2000; Takahashi et al., 2001; Ogiso et al., 2010). However, *Hd6* and *Hd16* were not likely to be involved in controlling the circadian clock. Interestingly, one non-synonymous alteration in Nipponbare-NIL (*Hd16*) shortens flowering time by 20 days under LDs and prolongs heading date by 3 days under SD, which is proved mostly because of the decreased phosphorylation of *Ghd7* (Hori et al., 2013; Kwon et al., 2014) (**Figure 6A**). Additionally, *Hd6* and *Hd16* also act upstream of *OsPRR37* and phosphorylate different regions of *OsPRR37* (Kwon et al., 2015). More important, *Hd6* and *Hd16* could also function as an enhancer of the bifunctional action of *Hd1*, and fine-tune the CDL of flowering (**Figure 6A**). Therefore, plants with functioning *Hd6* and *Hd16* show earlier flowering under 10 h day length, while delayed flowering with 14.5 h photoperiod (Nemoto et al., 2018).

### CCT family genes

The CONSTANS (CO), CO-LIKE, and TIMING OF CAB EXPRESSION1 (TOC1) domain-containing protein family (CCT), which was initially discovered in Arabidopsis thaliana, is involved in controlling flowering time (Putterill et al., 1995; Strayer et al., 2000). There are 41 CCT domain-containing genes in rice, and at least 18 have been linked to flowering control, including 6 dual-function flowering regulators: *Hd1*, *Ghd7*, *OsPRR37*, *OsCCT22*, *OsCCT38*, and *OsCCT41*. *OsCCT22*, *OsCCT38*, and *OsCCT41* suppress heading under LD and promote it under SD, indicating that they could enhance the photoperiod sensitivity of flowering in rice (Zhang et al., 2021) (**Figure 6B**).

### OsVIL1

A specific group of basic helix-loop-helix (bHLH) factors, which are known as phytochrome-interacting factor-like (PIL or PIF) family proteins, are important factors connecting light signal, phytochrome and downstream regulators (Yamashino et al., 2003). *OsLF* encodes an atypical HLH protein. By competing with OsPRR1 for interaction with Oryza sativa PHYTOCHROME-INTERACTING FACTOR-LIKE 13 (OsPIL13) and Oryza sativa PHYTOCHROME-INTERACTING FACTOR-LIKE 15 (OsPIL15) and repressing the expression of *OsGI* and *Hd1*, *OsLF* inhibits heading in rice **(** Zhao et al., 2011 **)**. Interestingly, Oryza sativa VERNALIZATION INSENSITIVE 3-LIKE 1 (OsVIL1), a component of Polycomb Repressive Complex 2 (PRC2) complex, could activate flowering by suppressing *OsLF* under SD and delay flowering by inducing *Ghd7* under LD, suggesting a relation between histone modification and flowering reversal pathways (Jeong et al., 2016) (**Figure 6C**).

## Conclusions and perspectives

The most consistent environmental characteristic on the earth is day length. Plants employ photoperiods to detect seasonal variations for making proper flowering time. In Arabidopsis, *CO* functions as a network hub to integrate numerous external and internal signals into the photoperiodic flowering pathway. Identifying and characterizing regulators that physically interact with CO and affect its activity are two important research advances (Shim et al., 2017). In rice, more than 80 flowering regulators have been identified, most of which are involved in the photoperiodic pathway. Thus, the photoperiod might be the most important flowering pathway in rice. As SDPs, rice has a CDL that fixes the schedule for flowering time, limiting the range of cultivation areas (Nemoto et al., 2018). Investigating the CDL control mechanism in rice requires thoroughly studying the functionally reversible gene.

*Hd1* is a homolog of *CO* and was the first bifunctional flowering regulator identified in rice. Numerous studies have shown that the direct effect of light on Hd1 protein complex activity but not the protein stability or transcription level is the primary determinant of photoperiodic flowering. Subsequently, other reversal regulators, such as *Ghd7*, *DTH8*, and *OsPRR37*, have proved to help rice judge photoperiod for flowering (Yano et al., 2000; Ishikawa et al., 2011). Interestingly, reversible factors are not found in more than 300 flowering regulators in Arabidopsis, suggesting a completely different mechanism for sensing day length between rice and Arabidopsis (Bouche et al., 2016). Revealing the molecular mechanism of CDL recognition in rice could unravel the nature of the difference in day length recognition in SDPs and LDPs. These molecular mechanisms could help us transform SD/LD crops into LD/SD crops at the genetic level to expand planting areas as needed to ensure food security.

Several issues need attention in future research to explore the reversal mechanism in the rice flowering pathway. First, there is still a key issue regarding the nature of Hd1 protein reversal activity. Is it a change in Hd1 protein activity due to phosphorylation or a functional inversion due to different protein complexes? What do light, dark, and photoperiod do to Hd1? We still lack the means to detect, for example, Hd1 site-specific phosphorylation antibodies and protein complexes with different functions where Hd1 is located in spatial and temporal features. Second, loss of *Hd1* function did not completely abolish rice’s critical day length setting in some background. As mutation of *Hd1* could not cause *OsPRR37* and *Hd16* to lose their flowering inversion function, other reversal mechanisms independent of *Hd1* in rice should be explored in the future. Third, many flowering genes have more than one interacting factor. Polygenic mutants construction is much more difficult and time-consuming than Arabidopsis, which makes genetic analysis more difficult. Fourth, according to our previous study, different bi-functional genes have different reverse CDLs (Sun et al., 2021). Although many bi-functional regulators have been reported, most studies did not examine their CDLs. Identifying these CDL regulation pathways will unravel the molecular mechanisms of CDL regulation in the future. Finally, though an atypical HLH protein OsLF was identified as a flowering regulator in rice, there is still less identification of physical or direct interacting regulators with phytochrome. Which genes play the role of PIL/PIF and to which downstream genes the day length signal transmitted, leading to the mechanism for CDL recognition, are far from fully understood.

## Author contributions

CS, CH, CZ, and XD wrote the paper. All authors contributed to the article and approved the submitted version.

## Funding

This study was supported by grants from the National Natural Science Foundation of China (32172022, 31371602, and 31401358) and Sichuan Science and Technology Program (2020YJ0408).

## Acknowledgments

All the figures were created with BioRender.com.

## Conflict of interest

The authors declare that the research was conducted in the absence of any commercial or financial relationships that could be construed as a potential conflict of interest.

## Publisher’s Note

All claims expressed in this article are solely those of the authors and do not necessarily represent those of their affiliated organizations, or those of the publisher, the editors and the reviewers. Any product that may be evaluated in this article, or claim that may be made by its manufacturer, is not guaranteed or endorsed by the publisher.
